# Auditory and visual connectivity gradients in frontoparietal cortex

**DOI:** 10.1002/hbm.23358

**Published:** 2016-08-29

**Authors:** Rodrigo M. Braga, Peter J. Hellyer, Richard J. S. Wise, Robert Leech

**Affiliations:** ^1^ Center for Brain Science Harvard University Cambridge Massachusetts; ^2^ Athinoula A. Martinos Center for Biomedical Imaging Department of Radiology Massachusetts General Hospital & Harvard Medical School Charlestown Massachusetts; ^3^ The Computational, Cognitive and Clinical Neuroimaging Laboratory Division of Brain Sciences Hammersmith Hospital Campus, Imperial College London London United Kingdom; ^4^ Centre for Neuroimaging Sciences Institute of Psychiatry, Psychology & Neuroscience, King's College London London United Kingdom

**Keywords:** auditory, visual, functional, structural, connectivity, gradients, frontoparietal cortex, tractograpy, resting‐state, functional magnetic resonance imaging

## Abstract

A frontoparietal network of brain regions is often implicated in both auditory and visual information processing. Although it is possible that the same set of multimodal regions subserves both modalities, there is increasing evidence that there is a differentiation of sensory function within frontoparietal cortex. Magnetic resonance imaging (MRI) in humans was used to investigate whether different frontoparietal regions showed intrinsic biases in connectivity with visual or auditory modalities. Structural connectivity was assessed with diffusion tractography and functional connectivity was tested using functional MRI. A dorsal–ventral gradient of function was observed, where connectivity with visual cortex dominates dorsal frontal and parietal connections, while connectivity with auditory cortex dominates ventral frontal and parietal regions. A gradient was also observed along the posterior–anterior axis, although in opposite directions in prefrontal and parietal cortices. The results suggest that the location of neural activity within frontoparietal cortex may be influenced by these intrinsic biases toward visual and auditory processing. Thus, the location of activity in frontoparietal cortex may be influenced as much by stimulus modality as the cognitive demands of a task. It was concluded that stimulus modality was spatially encoded throughout frontal and parietal cortices, and was speculated that such an arrangement allows for top–down modulation of modality‐specific information to occur within higher‐order cortex. This could provide a potentially faster and more efficient pathway by which top–down selection between sensory modalities could occur, by constraining modulations to within frontal and parietal regions, rather than long‐range connections to sensory cortices. *Hum Brain Mapp 38:255–270, 2017*. © **2016 Wiley Periodicals, Inc.**

## INTRODUCTION

Establishing which neural systems subserve the cognitive manipulation of sensory information remains a central question in neuroscience. Frontoparietal brain regions are recruited when attention is drawn to sensory stimuli [Carlson et al., 1998; Corbetta and Shulman, 2008; Hopfinger et al., 2000; Kastner et al., 1999; Shomstein and Yantis, 2006; Yantis et al., 2002]. Specifically, dorsal frontoparietal activity is increased during tasks that require attentional orienting to visual stimuli and locations [Corbetta et al., 2002]. Several studies have shown the existence of retinotopic maps in both dorsal frontal (frontal eye fields [FEF]) and dorsal parietal regions [superior parietal lobe (SPL); Moore and Armstrong, 2003; Ruff et al., 2008; Saygin and Sereno, 2008]. These findings provide strong evidence for a link between dorsal frontoparietal activity and visual processing.

The networks subserving the control of auditory information are less well understood. Frontoparietal activity has also been implicated in auditory attention [Bremmer et al., 2001; Bushara et al., 1999; Griffiths and Green, 1999; Griffiths et al., 1998; Krumbholz et al., 2009; Langner et al., 2011; Martinkauppi et al., 2000; Mayer et al., 2006; Pavani et al., 2002; Wu et al., 2007] even in the absence of visual stimuli [Maeder et al., 2001; Shomstein and Yantis, 2006; Sridharan et al., 2007]. However, it is unclear whether the same frontoparietal regions are recruited for both auditory and visual tasks. Several studies have reported activity in a dorsal frontoparietal network that overlaps with the visual system during auditory stimulus anticipation, processing and attention shifts [Bharadwaj et al., 2014; Downar et al., 2000; Krumbholz et al., 2009; Langner et al., 2011; Lee et al., 2012; Linden et al., 1999; Mayer et al., 2006; Shomstein and Yantis, 2004; Watkins et al., 2007; Wu et al., 2007]. In contrast, there is increasing evidence that visual and auditory attention are subserved by different networks [Braga et al., 2013b; Bushara et al., 1999; Degerman et al., 2006; Kong et al., 2014; Maeder et al., 2001; Salmi et al., 2007; Seydell‐Greenwald et al., 2014]. For example, Bushara et al. [1999] showed that while visual spatial localization recruits dorsal regions such as the SPL and FEF, auditory localization recruits ventral frontoparietal regions along the inferior parietal lobe (IPL) and inferior frontal gyrus (IFG). Recently, Kong et al. [2014] showed that auditory spatial attention recruits non‐retinotopic regions of the parietal lobe (PL). A more ventral frontoparietal network has also been implicated in the processing of sound movement [Griffiths and Green, 1999; Griffiths et al., 1998; Pavani et al., 2002] and spatial orienting [Bushara et al., 1999; Kong et al., 2014; Maeder et al., 2001; Salmi et al., 2007]. These findings raise the possibility that there is a differentiation of function along frontoparietal cortex, with dorsal regions being specialized for visual, and ventral regions being specialized for auditory processing.

Anatomically, tracer studies provide support for such a differentiation of function. The visual “where” pathway, implicated in visuospatial processes, is mediated by projections to dorsal parietal regions [Ungerleider and Mishkin, 1982; Yeterian and Pandya, 2010]. Lesions of these dorsal regions can lead to visuospatial deficits characteristic of hemispatial neglect [Mesulam, 1981; Parton et al., 2004]. In contrast, an auditory “where” pathway has been proposed that originates in more ventral post‐central regions along the superior temporal gyrus [Romanksi, 2007; Rauschecker, 1998; Kaas and Hackett, 1999]. These auditory and visual streams are thought to project to different, potentially interdigitated regions of the frontal lobe [Romanksi, 2007], with evidence from human neuroimaging that visually responsive frontal subregions sit superior to auditory subregions [Michalka et al., 2015]. Diffusion tractography studies have shown that parallel cortico‐cortical connections along the superior longitudinal fasciculi project from ventral and dorsal aspects of frontoparietal cortices [De Schotten et al., 2011; Szczepanski, 2013]. Language regions in the temporal and frontal cortices have been proposed to be connected by two ventral projections, one via the extreme capsule to the IFG, and one via the arcuate fasciculus to motor regions [Saur et al., 2008]. There is limited tractographic evidence for direct connections between auditory and superior frontal regions in humans [Jbabdi et al., 2013; Rilling et al., 2008]. The parietal cortices have also been divided into dorsal and ventral aspects based on differences in structural connectivity [Mars et al., 2011].

A dorsal–ventral separation of auditory and visual function might also be expected given inherent differences between stimulus processing in each modality. For example, visual processing requires saccade planning and eye movement control, functions which have been localized to dorsal frontoparietal regions particularly around the frontal eye fields [e.g., Nobre et al., 2000]. In language, ventral brain regions such as Broca's and Wernicke's areas are thought to be more specialized for auditory processes. Further, whereas visual stimuli are often consistent across time and require spatial orienting (a function which is also attributed to dorsal parietal regions), auditory stimuli are typically transient in nature and often indicative of an environmental change that elicits stimulus‐driven reorienting of attention (e.g., a phone ringing, the cocktail‐party effect). As such the different proposed roles of the dorsal and ventral attention networks in top–down and bottom–up orienting [Corbetta et al., 2002] might also be compatible with a dorsal–ventral separation of auditory and visual function.

If frontoparietal cortices do show a dorsal–ventral gradient in visual and auditory function, differences in the intrinsic strength of connectivity with auditory and visual cortices may be observable in frontoparietal cortex. In this study, we investigated whether frontoparietal cortices contain gradients of preferential functional and structural connectivity with auditory and visual cortices. The null hypothesis was that no differences in the relative connectivity with auditory and visual regions would be observable across prefrontal and parietal cortices. In contrast, the hypothesis was that prefrontal and parietal cortices would show a differential gradient in visual and auditory connectivity, specifically along the dorsal–ventral axis. Our results show strong dorsal–ventral gradients of preferential connectivity, with more dorsal regions favoring visual and more ventral regions favoring auditory cortices.

## METHODS

### Subjects

A group of 25 neurologically healthy subjects were scanned in a resting state for the functional connectivity (FC) study (12 male, age range 19–49, mean age 32.7 years). Subjects were recruited primarily from postgraduate students and staff at Imperial College London. No information was collected about the level of musical experience of the subjects. This data were previously used by Braga et al. [2013a] and Leech et al. [2012]. The study was approved by the Hammersmith and Queen Charlotte's and Chelsea Research ethics committee. For the structural connectivity analysis, the first ten subjects from the Human Connectome Project [Van Essen et al., 2013] were included (5 male, age range 22–35). The preprocessed version of the data was used [Glasser et al., 2013]. The number of subjects used in the diffusion data was limited to 10 due to the large computational load of the unconstrained probabilistic tractography analysis.

### Frontal and Parietal Seed Regions

Two seed ROIs covering the prefrontal and parietal cortices were produced by combining anatomical probability maps from the Harvard–Oxford Cortical Structural Atlas. For the prefrontal seeds, the superior, middle and inferior frontal gyri (pars triangularis and pars opercularis) atlas regions were combined. For the parietal seeds, the superior parietal lobule, angular gyrus, supramarginal gyrus (anterior and posterior division), and parietal operculum cortex atlas regions were combined. Atlas maps were thresholded at the 20% probability level (i.e., including only voxels that form part of that anatomical region in over 20% of the population) before being combined.

### Auditory and Visual Target Regions

Anatomical regions of interest (ROIs) were taken from the Harvard‐Oxford Cortical Structural Atlas. The left and right calcarine sulci (including primary visual cortex) and Heschl's gyri (including primary auditory cortex) were used as the target ROIs with which functional and structural connectivity was measured. Supporting Information Figures S1 and S2 show that the anatomical masks covered auditory and visual cortices in each subject. Early sensory ROIs were selected in order to maximize the separation of each sensory modality, which hypothetically would be harder if associative and secondary sensory ROIs were used. We also repeated the FC analysis using larger data‐derived networks as target ROIs. The auditory network and dorsal and ventral visual streams network were manually selected from a whole‐brain ICA of the functional data (20 dimensions, 25 subjects, see Functional connectivity analysis description) and used as the functional ROIs. This confirmatory analysis yielded qualitatively similar results to the more focused analysis using ROIs based on approximate anatomical locations of early sensory cortices; however, it suffered from the potential confound that the higher‐order processing streams project into the parietal lobe. As such, to keep the parietal connectivity analysis anatomically separated from the target ROIs, the anatomical ROIs were used for the quantitative assessment of auditory and visual connectivity bias.

The null hypothesis in this experiment relates to the functional connectivity of parietal and frontal lobe regions with a set of target voxels in separate non‐overlapping locations: the occipital and temporal lobes. The spatial‐ICA (which was the alternative way to define target ROIs) was used to localize in a data‐driven manner the occipital and temporal voxels that showed intrinsic functional connectivity with each other (consistent with extended visual and auditory cortical systems). The ICA was not used to define the parietal or frontal ROIs. Although the spatial‐ICA was performed on the same data as the subsequent functional connectivity, the definition of source ROIs (voxels in frontal or parietal regions, selected anatomically) was not informed by the definition of the target ROIs (occipital or temporal, defined functionally). In addition, the voxels involved in the ROIs were masked and hence non‐overlapping. This was done to avoid bias in the results of the FC analysis.

### fMRI Data Acquisition

MRI data were obtained using a Philips (Best, The Netherlands) Intera 3.0 Tesla MRI scanner using Nova Dual gradients, a phased‐array head‐coil, and sensitivity encoding (SENSE) with an under‐sampling factor of 2. Functional MRI images were obtained using a T2*‐weighted gradient‐echo echoplanar imaging (EPI) sequence with whole‐brain coverage (TR/TE = 2,000/30; 31 ascending slices with thickness 3.25 mm, gap 0.75 mm, voxel size 2.19 × 2.19 × 4 mm, flip angle 90°, field of view 280 × 220 × 123 mm, matrix 112 × 87). Quadratic shim gradients were used to correct for magnetic field inhomogeneities within the brain. T1‐weighted whole‐brain structural images were also obtained in all subjects for registration. Three hundred volumes were acquired while subjects lay in the scanner with their eyes closed.

### fMRI Data Preprocessing

EPI images were realigned to the middle volume in the fMRI data series for each subject to remove the effects of motion between scans using FSL's MCFLIRT [Jenkinson et al., 2002]. Spatial smoothing was performed using a 6 mm full‐width half‐maximum Gaussian kernel, and pre‐whitening with FILM and temporal high‐pass filtering using a cut‐off frequency of 1/50 Hz to correct for baseline drifts in the signal. FMRIB's Linear Image Registration Tool [FLIRT; Smith et al., 2009] was used to register EPI functional datasets into standard MNI space using the participants' individual high‐resolution anatomical images (12 d.o.f). In addition, variance associated with motion (6 variables), and the time series of white matter and cerebrospinal fluid were removed from the whole brain functional data using ordinary least squares linear regression. Mean time series were extracted from a 3 mm‐radius sphere within the white matter (MNI −26, −22, 28) and from one lateral ventricle (MNI 2, 10, 8). Average whole brain or gray matter activity was not regressed so as not to complicate the interpretation of negative functional correlation findings [Murphy et al., 2009]. The fMRI data was downsampled by a factor of 2 prior to the functional connectivity analysis to reduce computational load.

### Functional Connectivity Analysis

**Figure 1:**
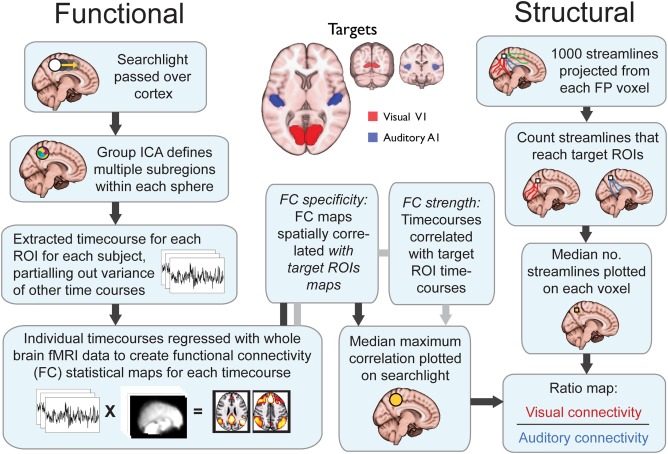
Schematic of pipeline for relative auditory and visual structural and functional connectivity analysis.

Figure 1 shows a schematic of the analysis steps. FC was tested using a previously described multivariate method [Braga et al., 2013a; Leech et al., 2012] using FMRIB's Software Library [FSL v4.1; http://www.fmrib.ox.ac.uk/fsl; Smith et al., 2009] and in‐house analysis scripts. Briefly, a spatially restricted independent component analysis [ICA; MELODIC; Beckmann et al., 2005] was used to decompose the BOLD signal from within a 16 mm‐diameter (251 voxel) spherical searchlight into 10 spatial components as described in detail in [Braga et al., 2013a]. Searchlights were passed across the whole brain, but for clarity and consistency with our hypothesis in the present analysis the results were restricted to the prefrontal and parietal cortices by post‐hoc masking. Dual regression was used to reveal the whole‐brain FC patterns of these 10 components [Leech et al., 2012; Braga et al., 2013a, 2013b; Braga and Leech, 2015] as follows. Briefly, a time series was extracted from each of the 10 components in a spherical ROI (first regression). The whole‐brain FC maps corresponding to these time series were then calculated (second regression). In the first regression, the 10 spatial component maps were included as the explanatory variables (EVs) and regressed against each subject's 4D functional data (considering searchlight voxels only). In the second regression, these time series were again entered as the EVs in a regression against each subject's 4D functional data, this time considering voxels across the whole brain. This produced a subject‐specific whole‐brain FC map for each of the 10 searchlight time series. To test for FC with auditory and visual regions, the resulting 10 whole‐brain FC maps obtained from each searchlight were spatially correlated with the auditory and visual target ROI maps. Bilateral auditory and visual target ROIs were used as targets because the primary sensory regions often show highly correlated activity with their contralateral counterparts even at rest [e.g., Smith et al., 2004].

This multivariate analysis pipeline produces multiple FC patterns from each searchlight. Our previous work has shown that heteromodal regions such as those found in frontoparietal cortices contain hidden signals. These hidden signals are not adequately revealed using univariate FC techniques, which are based on the average signal within a region [Braga and Leech, 2015; Braga et al., 2013a; Leech et al., 2012]. The multivariate approach used therefore allowed us to measure the specificity of connectivity of frontoparietal regions with both auditory and visual targets even in instances where the connectivity with one target dominates the average change in fMRI signal. To compute a measure of relative FC (F_VA_), the maximum spatial correlation between the 10 searchlight FC maps and each target ROI was taken for each subject. Across subjects, the median of these maximum spatial correlations was then taken and plotted as a single value on to the searchlight. At locations where two or more searchlights overlapped, the average value across the searchlights was taken. The distribution of “median maximum correlation values” with visual and the auditory targets was then compared (visual connectivity divided by auditory connectivity; V/A) to produce a ratio map of relative functional specificity. Values close to 0 represent preferential connectivity for auditory cortex, while values higher than 1 represent preferential connectivity to visual cortices.

As the FC measure described above is derived from a spatial correlation, it does not arguably represent the FC *strength*, but rather the *specificity* of the searchlight's subsignals for visual‐ and auditory‐cortices. For example, even if a target and seed were strongly functionally correlated with each other, if they were also functionally correlated with a third region then the seed's FC map would show lower spatial correspondence with the target. To support the above FC analysis, we also tested the FC *strength* between target and seed regions. The 10 time series from each searchlight were regressed against the average signal time series from the seed ROIs. This measured the temporal, as opposed to spatial, correlation between the seed and targets. As with the spatial correlation analysis, at each searchlight, the maximum temporal correlation was taken for each subject, and across subjects the median maximum value was taken and plotted in each searchlight. This analysis produced a qualitatively similar distribution of auditory–visual FC bias to the spatial correlation analysis, therefore only the spatial correlation results are presented in detail below.

### Diffusion Data Acquisition

Diffusion MRI (dMRI) data from the Human Connectome Project were obtained using a Siemens “Connectome Skyra” 3.0 Tesla MRI scanner using a 32‐channel receive head‐coil, and a customized SC72 gradient insert [Uğurbil et al., 2013]. Diffusion weighted MRI images were obtained using a spin‐echo echoplanar imaging (EPI) sequence with whole‐brain coverage (TR/TE = 5,520/89.5; 111 ascending slices with thickness 1.25 mm, voxel size 1.25 × 1.25 × 1.25 mm, flip angle 78°, field of view 210 × 180 mm, matrix 168 × 144, with a multiband acceleration factor of 3). dMRI acquisition was acquired in 6 runs of approximately 10 min representing three different diffusion tables with 90 diffusion directions acquired in both right to left and left to right phase encoding directions. Diffusion weighting was organized into three different shells of *b* = 1,000, 2,000, and 3,000 s/mm^2^.

### Structural Connectivity

Structural connectivity was tested using high angular resolution diffusion imaging (HARDI) and probabilistic tractography. Preprocessed versions of the data from the Human Connectome Project [Glasser et al., 2013] were used. In brief, EPI distortion was corrected within raw dMRI data, using TOPUP followed by eddy correction using EDDY from the FMRIB software library [Smith et al., 2009]. Diffusion data and diffusion direction information were then registered to an individual subject T1 image, using boundary‐based registration of the b0 image. Tractography was performed using FMRIB's Diffusion Toolbox (FDT v2.0) as implemented in FSL. Firstly, we modeled the probability distribution of fiber direction within each voxel using FSL's BedpostX in order to account for crossing fibers. Probabilistic tractography was carried out using FSL's ProbtrackX [Behrens et al., 2003].

Figure 1 shows a schematic of the analysis steps. One thousand streamlines were projected from each voxel within the seed ROIs. Between pairs of voxels, the default curvature threshold of 0.2 was used to discard streamlines which made sharp turns. No waypoint or termination ROIs were used to constrain the streamline distributions. At every voxel, the number of streamlines that reached the target auditory and visual ROIs were counted. The number of streamlines reaching the visual target was then divided by the number of streamlines reaching the auditory target (V/A) to compute an index of relative structural connectivity (S_VA_). Values close to 0 represent connectivity weighted toward auditory cortex, while values higher than 1 represent a weighting toward visual cortices. In the structural connectivity analysis, the connectivity from seed ROIs to left and right targets was assessed independently. Within each modality (auditory, visual), the left and right connectivity maps were then averaged together to produce a single map for the bilateral targets.

### Gradient Analysis

To quantify the extent to which spatial gradients can be found in lateral frontoparietal cortices, the connectivity ratio images (F_VA_ and S_VA_) were projected onto a 2‐dimensional plane by taking the average along the x (medial to lateral) axis. This was done for each hemisphere independently. The medial voxels (7 voxels from the midline) were discarded so that the lateral, not medial, prefrontal, and parietal cortical surface could be studied. The natural logs of all ratios were calculated before the distributions were entered into a linear regression. A design matrix containing the distance in voxels along horizontal (posterior to anterior) and vertical (inferior to superior) directions was produced for both the parietal and prefrontal cortices. These vertical and horizontal gradient images were then both regressed against either the F_VA_ or S_VA_ log ratio images using ordinary least squares linear regression. This allowed us to test how much of the F_VA_ and S_VA_ patterns could be explained by the two linear gradients. To test whether there were inter‐hemispheric differences in gradient strength, the log ratio images for left and right hemispheres were contrasted (right minus left), disregarding voxels for which there was no contralateral equivalent. The resulting subtraction image was entered into a regression with the horizontal and vertical distance as explanatory variables. All *P*‐values were corrected for multiple comparisons using Bonferroni correction for the different connectivity distributions tested (S_VA_, F_VA_, PFC and parietal cortex, right and left hemisphere, with and without covariate regression), unless otherwise stated.

### Controlling for Distance

In tractography, the further that two voxels are from each other the less likely they are to be connected by a streamline, irrespective of the true anatomical connections. Therefore, it is possible that the relative distance of a voxel from the auditory and visual cortices could determine the pattern of relative structural connectivity observed. To control for this, a map of relative distance to each target at each voxel (i.e., the Euclidian distance between that voxel and the visual ROI divided by the distance between that voxel and the auditory ROI) was produced. This map was entered as an explanatory variable and regressed against the S_VA_ map using ordinary least squares regression. The residual image from this regression, therefore, showed the structural connectivity bias after controlling for relative distance to the targets. In addition, to assess whether the relative FC pattern was determined solely by the structural connectivity pattern, the S_VA_ map was regressed against the F_VA_ map. The residuals of this regression represented FC asymmetries that could not be explained by the relative (direct) structural connections, as measured by tractography. We also investigated if motion (mean frame wise displacement per subject) and age were related to individual measures of vertical or horizontal gradients. We observed no significant relationship of motion or age with either vertical or horizontal gradients on either the left or right in either the PFC or PL (all *P*‐values >0.1).

## RESULTS

### Structural Connectivity Gradients

**Figure 2:**
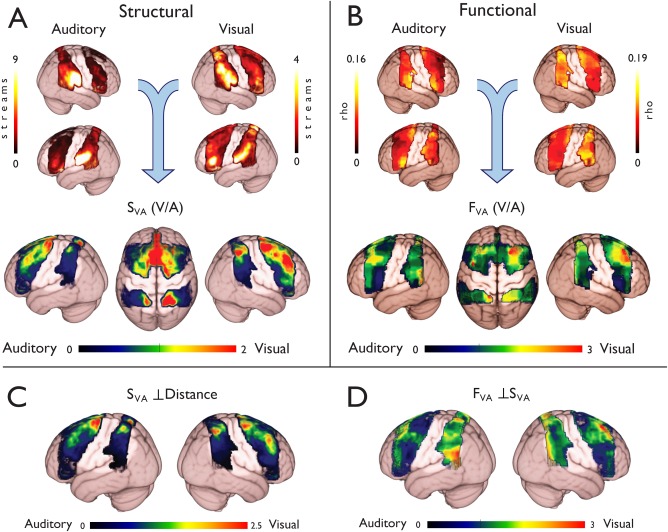
Visual–auditory connectivity differences in frontoparietal cortex. (A) Structural connectivity was assessed by counting the number of probabilistic tractography streamlines (“streams”) that reached the auditory and visual targets cortices. Relative structural connectivity was assessed by comparing the visual and auditory maps (V/A; S_VA_). This analysis revealed marked connectivity differences, where dorsal regions were biased toward visual connections, while ventral regions showed an auditory bias. (B) Functional connectivity was assessed using multivariate methods (see Fig. 1). A similar dorsal to ventral gradient was observed in the ratio image (F_VA_), with dorsal regions favoring visual, and ventral regions favoring auditory targets. (C) The relative Euclidian (straight line) distance to the targets was regressed out of the structural data to test for a potential confound. This analysis still revealed distinct foci of visual and auditory bias in dorsal and ventral regions respectively. (D) The structural results were regressed out of the functional results to test whether the structural pattern could explain the observed functional biases. Residual functional connectivity differences were observed in both prefrontal and parietal cortices.

Both prefrontal and parietal cortices showed differences in their structural connectivity with auditory and visual cortices (Fig. 2A). Bilateral IPL regions near the temporoparietal junction showed the strongest connectivity with auditory regions. The bilateral IPL also showed strong structural connectivity to visual cortices, but similar levels were observed in dorsal parietal regions, particularly in the right SPL. Strong connectivity was also observed between the left inferior frontal gyrus and visual regions.

**Figure 3:**
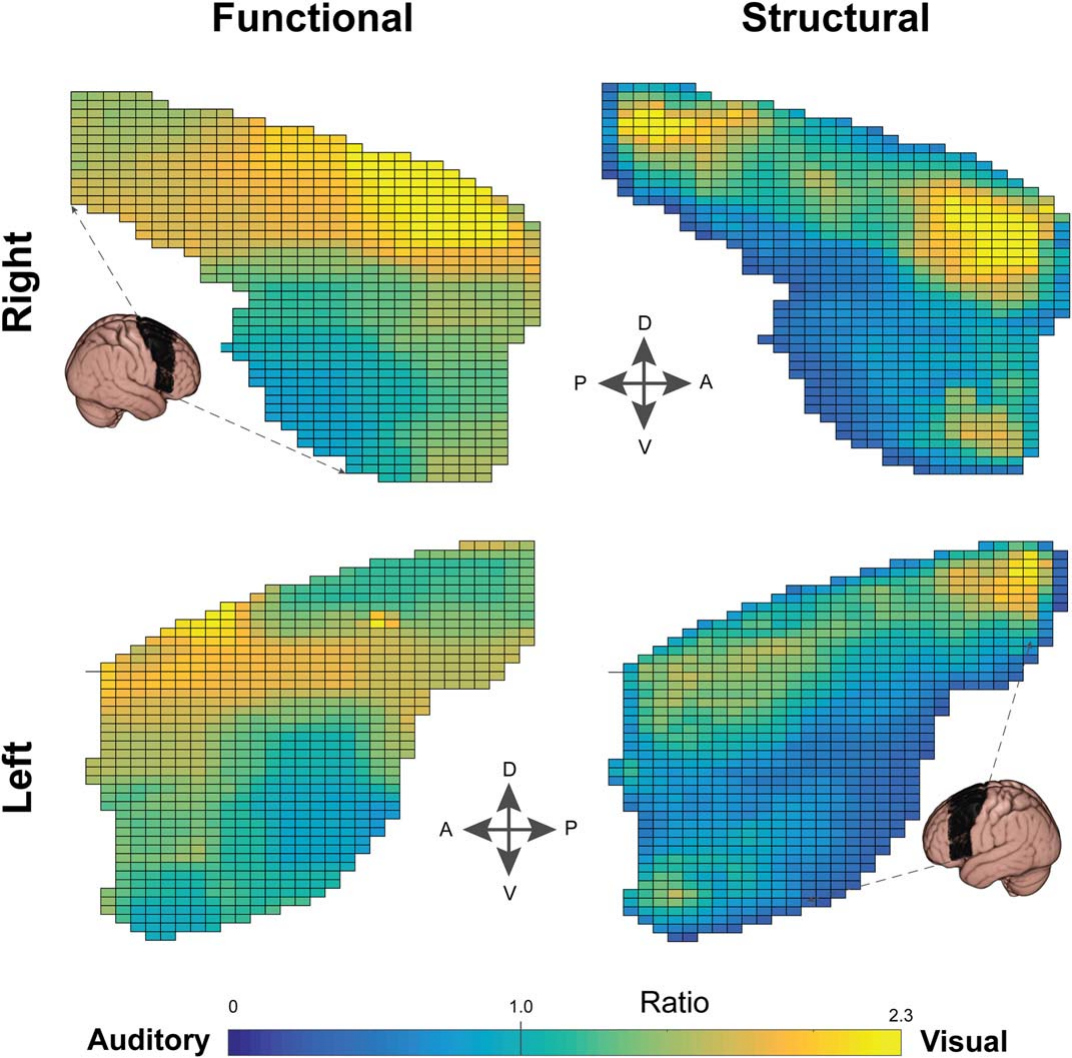
Connectivity gradients in prefrontal cortex. Functional and structural connectivity analyses both revealed a graded transition across the prefrontal cortices. In general, dorsal regions favored visual and ventral regions favored auditory targets. In this figure, connectivity values along the medial‐lateral axis were projected onto the 2D grid displayed; therefore each grid element represents the average of a vector of voxels.

**Figure 4:**
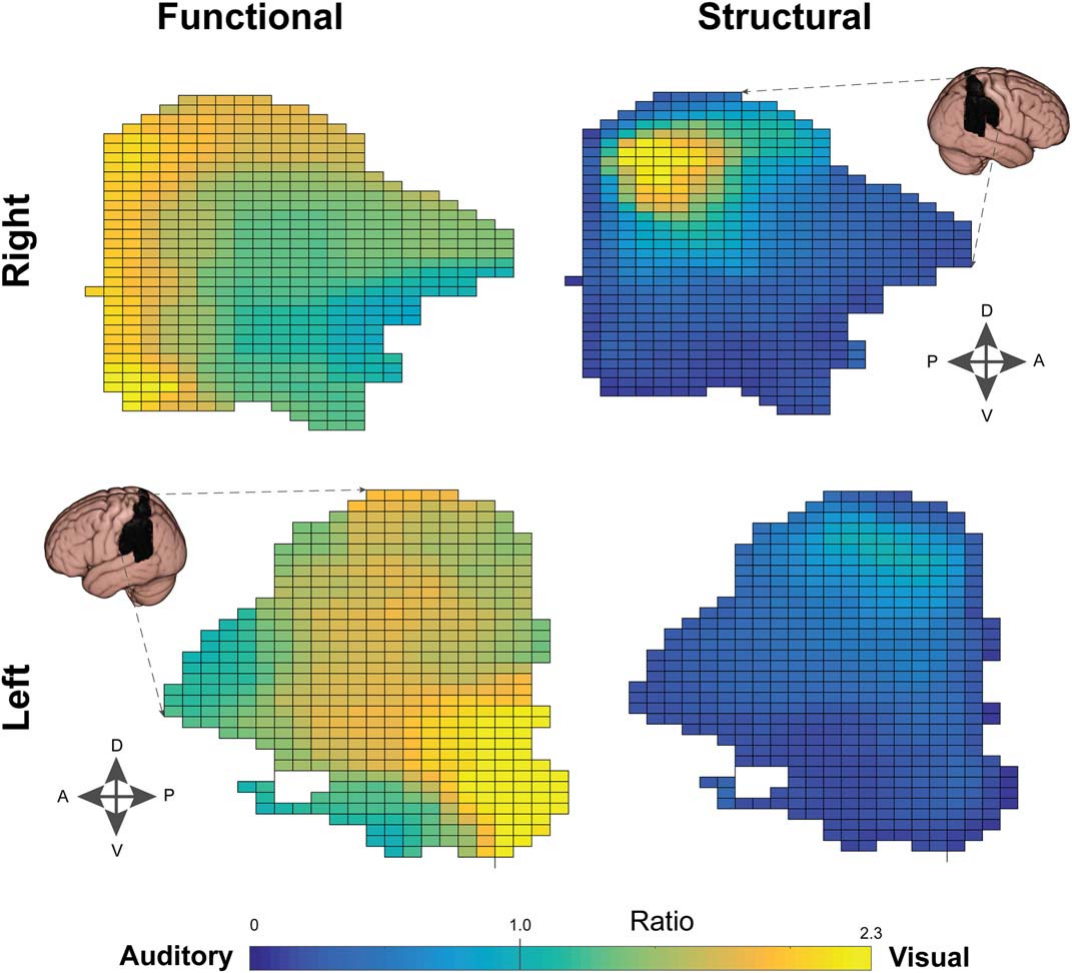
Connectivity gradients in parietal cortex. Structural gradients followed a dorsal–ventral transition similar to the functional results (see Fig. 3) with a locus of visual bias in SPL. Functional gradients were more complex, with evidence for a posterior–anterior (as well as dorsal–ventral) gradient in the right hemisphere. In contrast, the left parietal lobe showed a posterior–anterior but not dorsal–ventral gradient. In this figure, connectivity values along the medial–lateral axis were projected onto the 2D grid displayed; therefore each grid element represents the average of a vector of voxels.

When the visual and auditory connectivity maps were compared (i.e., the ratio of visual to auditory structural connections; S_VA_), the pattern of *relative* structural connectivity became clearer (Figs. 2A, 3, and 4). Overall the image was skewed toward auditory connectivity, with a median value of 0.63 (Quartiles: Q1 = 0.35, Q3 = 1.28), where a value of 1 represents equal weighting toward visual and auditory cortices. Despite of this, dorsal regions of both the frontal and parietal cortices appeared to be more strongly biased toward visual than auditory targets, whereas inferior frontoparietal regions showed stronger auditory than visual connectivity.

**Figure 5:**
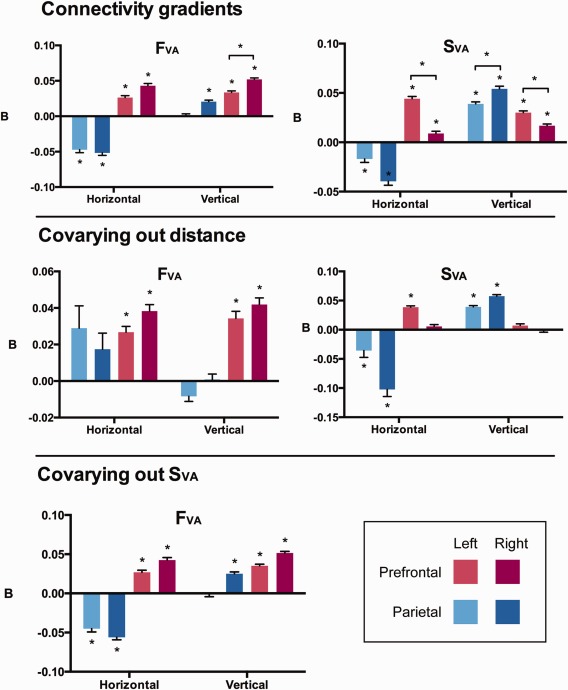
Gradient regression results. To quantify the imaging results, a linear regression was used to test whether the position of each voxel along a horizontal (anterior to posterior) and vertical (ventral to dorsal) axis explained the structural and functional connectivity patterns (top row). Functional and structural gradients were strongly detected in the prefrontal cortex (red colors). These gradients represented a visual–auditory transition in the dorsal–ventral and posterior‐anterior directions, respectively. In the parietal lobe (blue colors), similar structural gradients were observed in the dorsal–ventral direction. However in the horizontal axis the gradient followed an opposite pattern to the prefrontal cortex. We also repeated the analysis while covarying out the relative distance to targets (middle row), and covarying out the structural results (S_VA_) from the functional data (lower row).

To quantify the observations reported above (which are essentially descriptive), we performed a regression analysis using horizontal and vertical position as explanatory variables (Figs. 3, 4, 5). Both the prefrontal (Right: *t*_974_ = 9.46; Left: *t*_1071_ = 16.61; both *P* < 0.0001) and parietal cortices (Right: *t*_598_ = 21.05; Left: *t*_566_ = 18.5; both *P* < 0.0001) showed evidence for a “vertical” (i.e., dorsal‐ventral) structural gradient, with more dorsal regions favoring visual cortex (Fig. 5). This gradient was more pronounced in left PFC than the right (*t*_974_ = −7.38, *P* < 0.0001), and conversely, was stronger in the right PL than the left (*t*_566_ = 24.71, *P* < 0.0001). In the “horizontal” (i.e., posterior to anterior) axis the PL displayed a visual to auditory structural gradient (Right: *t*_598_ = −9.60; Left: *t*_566_ = −4.78; both *P* < 0.0001), while prefrontal cortices displayed an auditory to visual transition (Right: *t*_974_ = 3.82, Left: *t*_1071_ = 20.28, both *P* < 0.0001) gradients, respectively. The horizontal gradients were much more pronounced in left PFC than the right (*t*_974_ = −20.96, *P* < 0.0001), but no differences were observed between left and right PL (*t*_566_ = −0.95, *P* = 0.25).

To account for the possible confound of relative distance to the targets, the relative Euclidian distance was regressed out of the S_VA_ map (Fig. 2C). This analysis revealed a similar pattern to the S_VA_ map, with a dorsal–visual to ventral–auditory pattern which was perhaps even more pronounced. Once the relative Euclidian distance to each target was covaried out, the image became more skewed toward auditory connectivity (median = 0.52, Q1 = 0.21, Q3 = 1.13). However, there was still evidence of a vertical gradient in the parietal (Right: *t*_597_ = 22.22; Left: *t*_565_ = 18.63: both *P* < 0.0001) but not prefrontal cortices (Right: *t*_973_ = −0.35, *P* = 0.38; Left: *t*_1073_ = 2.38, *P* = 0.02 uncorrected; Fig. 5). Evidence for a residual horizontal gradient was also observed in the parietal cortex (Right: *t*_597_ = −8.38, *P* < 0.0001; Left: *t*_565_ = −3.12, *P* < 0.005) and left PFC (Left: *t*_1073_ = 17.77; *P* < 0.0001), but not right PFC (*t*_973_ = 2.43, *P* = 0.02 uncorrected).

### Functional Connectivity Gradients

In the right hemisphere, ventral parietal and frontal regions (around the IPL and IFG) showed higher FC specificity for bilateral auditory targets (by eye; Fig. 2B, top panel). In the left hemisphere, the IFG also showed stronger connectivity with auditory regions, but dorsal regions of the PL were more specific for auditory cortex than ventral regions (by eye; Fig. 2B, top panel). There was also evidence of increased auditory connectivity in left dorsal regions along the bilateral posterior superior frontal gyrus (SFG; near the FEF), compared with neighboring middle frontal gyrus and the rostral aspects of the PFC ROI. Dorsal PFC regions also showed stronger connectivity with visual cortex than ventral PFC, particularly in the right hemisphere. In the PL, asymmetries in visual connectivity were harder to identify, with both dorsal and ventral regions showing strong visual connectivity.

Differences in the auditory–visual pattern of connectivity were easier to identify when the relative connectivity strength (Ratio: visual/auditory; F_VA_) was assessed (Fig. 2B, lower panel). Generally, the ratio image was skewed toward visual connectivity, with a median value of 1.36 (Quartiles: Q1 = 1.15, Q3 = 1.65). Right dorsal prefrontal regions showed a bias toward visual cortices, while ventral regions favored auditory cortex (Fig. 3). In the PL, a superior–inferior pattern was also evident (Fig. 4).

These observations were supported quantitatively by the regression analysis of the functional data (F_VA_; Fig. 5). The prefrontal cortices showed evidence for a “vertical” (i.e., dorsal–ventral) gradient (Right: *t*_239_ = 25.92; Left: *t*_302_ = 16.06, both *P* < 0.0001), with more dorsal regions favoring early visual cortex. This gradient was more pronounced in the right PFC than the left (*t*_159_ = 9.17, *P* < 0.0001). There was also evidence for a “horizontal” (i.e., posterior–anterior) gradient (Right: *t*_239_ = 13.31; Left: *t*_302_ = 10.14; both *P* < 0.0001), with more posterior regions showing stronger auditory connectivity. In the PL, evidence for a posterior–anterior gradient was also found in the opposite direction (Right: *t*_175_ = −14.89; Left: *t*_172_ = −11.38; both *P* < 0.0001). Evidence for a “vertical” dorsal–visual to ventral–auditory gradient was also observed in the right (*t*_175_ = 9.10, *P* < 0.0001), but not left (*t*_172_ = 0.34, *P* = 0.38), parietal cortex.

To investigate whether these connectivity gradients were determined largely by the structural connectivity pattern, we regressed out the S_VA_ from the F_VA_ map. In the right dorsal PFC, along middle and superior frontal gyri, there was evidence of a residual visual bias (Fig. 2D). Evidence for a residual “dorsal–ventral” gradient was observed in the prefrontal cortex (Right: *t*_238_ = 25.67; Left: *t*_301_ = 16.92; both *P* < 0.0001; Fig. 5) and right, but not left, parietal cortex (Right: *t*_174_ = 11.04, *P* < 0.0001; Left: *t*_171_ = −0.50, *P* = 0.35). A horizontal residual gradient was also observed in both prefrontal (Right: *t*_238_ = 13.21; Left: *t*_301_ = 10.61; both *P* < 0.0001) and parietal (Right: *t*_174_ = −16.79; Left: *t*_171_ = −10.63; both *P* < 0.0001) cortices, but in opposite directions. In parietal cortex, more posterior regions were strongly connected to visual cortices while in prefrontal cortices more anterior regions had stronger visual connectivity.

We also tested whether the F_VA_ pattern could be explained by the relative distance to the targets by including the Euclidian distance variable in the regression. A dorsal–ventral gradient remained in the PFC (Right: *t*_238_ = 11.69; Left: *t*_301_ = 8.73; both *P* < 0.0001) but not consistently in the PL (Right: *t*_174_ = 0.28, *P* = 0.38; Left: *t*_171_ = −3.04; *P* < 0.005 uncorrected for multiple comparisons). Similarly, in the posterior–anterior axis a gradient was observed in the PFC (Right: *t*_238_ = 11.10; Left: *t*_301_ = 8.67; Both *P* < 0.0001) but not strongly in the PL (Right: *t*_174_ = 1.99, *P* = 0.0556; Left: *t*_171_ = 2.37, *P* = 0.0247, uncorrected).

## DISCUSSION

In this study, we show that frontal and parietal cortices exhibit striking differences in their relative connectivity with auditory and visual cortices. A spatial gradient was observed (at the macroscopic scale), where dorsal frontoparietal regions displayed greater connectivity with early visual areas, while ventral regions were weighted toward auditory connections. Importantly, these differences were most striking in the relative likelihood of white matter structural connections (Figs. 2A and 5). A complementary analysis of functional neuroimaging data revealed a similar dorsal‐ventral gradient using resting‐state FC (Figs. 2B and 5), particularly in the right hemisphere. The presence of these dorsal–ventral gradients may represent a fundamental organizational principle of frontoparietal cortex.

The frontoparietal cortices have been divided into dorsal and ventral subdivisions in the context of task‐driven demands. A prominent theory is that dorsal regions are activated for visual top–down attention, while ventral regions are driven by stimulus properties [Corbetta et al., 2002]. There is controversy over whether this same pattern is generalizable to non‐visuospatial tasks [Alho et al., 2015; Braga et al., 2013b; Gottlieb and Snyder, 2010; Kong et al., 2014; Salmi et al., 2009]. It is unclear whether the same frontoparietal set of putative “multimodal” regions is activated during the cognitive processing of information from all sensory modalities. However, a dorsal–ventral split between visual and auditory regions has been hypothesized previously based on both cognitive neuroimaging and histological evidence [Braga et al., 2013b; Bushara et al., 1999; Kong et al., 2014; Rauschecker and Scott, 2009; Romanski, 2004]. In this study, we provide evidence that this distinction is detectable in humans using both structural and task‐independent functional imaging. This suggests that the location of frontoparietal activation during a cognitive task is likely to be affected by intrinsic biases toward auditory or visual inputs. As such, more dorsal frontoparietal activity may be more concerned with visual processing, while ventral frontoparietal activity may be more related to auditory processing. The visual/auditory distinction is unlikely to involve a hard discontinuity; our statistical analysis suggests that there exists a graded transition in relative sensory preference across lateral prefrontal and parietal cortex. This graded transition is therefore not incompatible with prior findings of overlapping frontoparietal activity during auditory and visual tasks [e.g., Martinkauppi et al., 2000].

Our findings are broadly consistent with current theories of frontoparietal function. For example, speech production and comprehension is typically regarded as a function of ventral prefrontal regions such as Broca's area [Vigneau et al., 2006], while eye movement and saccade planning are isolated to the dorsal frontoparietal cortices [Hu and Walker, 2011; Isoda and Tanji, 2003]. In relation to the dorsal and ventral attention network theory [Corbetta and Shulman, 2008], the idea that visuospatial top–down attention is localized to the dorsal frontoparietal cortex agrees with our findings of dorsal visual dominance. It is less clear how the proposed role of the ventral attention network in stimulus‐driven (bottom‐up) attention can be reconciled with the auditory function observed ventrally here. Temporoparietal and inferior/middle prefrontal cortices have been implicated in auditory “change‐detection” [Doeller et al., 2003; Tse et al., 2006], and auditory effects have been observed in the ventral attention network [Mayer et al., 2006; Watkins et al., 2007]. It has been hypothesized that the activation of the ventral attention network represents a “reorienting response” [Corbetta et al., 2002], which mediates the reorienting of the senses to the changed stimulus via transient activity in ventral regions. This reorienting of attention could be an intrinsically multimodal process, so that all senses can be brought to focus on the relevant, changed stimulus. This is supported by findings that crossmodal stimuli can affect performance on a visual task [Driver and Spence, 1998]. Thus activity in ventral auditory‐biased frontoparietal regions during reorienting may represent a refocusing or inhibiting of auditory processes due to visual attention capture.

The relevance of these large‐scale, gradual connectivity changes is yet to be determined. Although some ventral frontoparietal regions are closer (spatially) to the auditory cortex, our analyses suggest that distance alone is unlikely to explain the patterns observed. In addition, the fact that similar results were observed using both diffusion‐tractography and functional MRI suggests that the observed patterns are unlikely to have been introduced by the choice of imaging methodology. Rather, such a graded transition could represent a mechanism by which the higher‐order segregation and integration of sensory information is achieved. For example, when an object is perceived through both visual and auditory senses, top–down attentional signals might need to modulate either visual or auditory stimulus properties, or both. If the source (auditory or visual) of those properties is also spatially encoded in higher‐order regions, a more efficient route for top–down control could be achieved by constraining the modulations to the local frontoparietal topography. The alternative would be to have long‐range inhibitory and facilitatory connections projecting to early sensory cortices. This alternative may be costly in metabolic terms and in the time taken for information to be transmitted. Previous work has shown that frontoparietal connections are likely to be reciprocal, and thus could represent both ascending and descending modulatory pathways [Pandya and Kuypers, 1969; Petrides and Pandya, 1984].

Although a large‐scale gradient was observed in both frontal and parietal lobes at the macroscopic level, it is not clear whether auditory or visual function is still mediated by specific subregions which were beyond the resolution of the current methodology. The functional analysis used large ROIs, and could only reveal large‐scale (and gradual) changes in auditory–visual preference. Therefore, it is possible that the present results are due to local interspersed auditory and visual modality‐specific subregions [e.g., Michalka et al., 2015], which become more frequent for a given modality during the transition from dorsal to ventral regions. As such, the graded transition may not necessarily be a consequence of polymodal neurons exhibiting relative modality preferences. However, the FC results were confirmed by the tractography analyses, which was performed voxel‐wise (rather than using ROIs). Although this method is not without its own caveats, it corroborates the finding that there is some macroscopic differentiation of sensory function along frontoparietal cortices. In addition, it is likely that there are interesting inter‐individual differences in the level of functional specificity (e.g., as a consequence of level of musical training, bilingualism or age) which the present analysis was not designed or sufficiently powered to test. These might yield to a more comprehensive future analysis.

It is not clear how the observed gradients may affect or contribute to multimodal spatial maps in the PL. Speculatively, the presence of neighboring connections between IPL and SPL may allow auditory spatial information (e.g., from the auditory “where” pathway) to elicit activation in the visual “where” pathway in the SPL [Romanski, 2004]. Similarly, in the PFC, lateral connections may allow the selection of information from a given modality while inhibiting nearby regions subserving other modalities. Surround inhibition is observed in the frontal lobe during stimulation of a visual receptive field [Cavanaugh et al., 2012]. Although on a different spatial scale, we have previously observed the relative deactivation of ventral PFC during a visual attention task, while conversely, dorsal PFC regions were deactivated during an auditory attention task [Braga et al., 2013b].

The structural connectivity pattern was largely similar to the functional pattern, showing a broad dorsal‐ventral gradient. However, when the structural pattern was regressed out of the functional data, residual functional asymmetries were observed in the right PFC and left PL (Fig. 2D). This suggests that there are intrinsic differences in the FC biases that are not mediated by direct, major white‐matter connections. The right dorsal PFC regions that showed residual connectivity were near the superior frontal gyrus and sulcus, and extended into middle frontal gyrus; regions which make up the frontal part of the “dorsal attention network” and are implicated in visual attention [Corbetta et al., 2002]. One possibility is that these functional biases are mediated by indirect (e.g., thalamocortical) projections. Alternatively, the relative strength of myelination of white matter fibers might explain the observed FC biases not accounted for by the number of connecting streamlines. The residual biases could also be the result of the visual and superior frontal regions being functionally connected through a third cortical source. However, as the specificity of FC maps for target ROIs was assessed, this is less likely to be the case. It is interesting that the FC distribution was skewed toward visual cortices, while the structural connectivity was skewed toward auditory cortices. This may be for several reasons, such as an interaction between the size of the ROI (the visual ROI was larger than the auditory ROI) and the distance from seed voxel to ROI (the auditory ROI was generally closer to the PFC seeds). It is also possible that the visual FC results are more mediated by indirect connections (e.g., thalamocortical projections) than the auditory FC results. These factors could each have a different influence on the results from each imaging technique.

In the left supramarginal gyrus (SMG), strong residual FC with visual cortices was observed after the structural pattern was accounted for (Fig. 2D). This suggests that, despite a dominant anatomical link to neighboring auditory centers (Fig. 2), the left SMG still shows strong FC with visual cortices. This agrees with the hypothesized role of the SMG as a multimodal integration region [Downar et al., 2000; Mesulam, 1998]. Structurally, this residual FC bias may be a result of neighboring cortico‐cortical connections which were not considered by the tractography algorithm due to sharp turns.

Auditory–visual gradients were also observed in the posterior–anterior (“horizontal”) axis (Fig. 5). In agreement with a recent fMRI study [Mayer et al., 2016], anterior PFC regions were more connected to visual cortices, while posterior PFC regions were more auditory (Fig. 3). Conversely, posterior PL regions were more strongly visual, while more anterior PL regions were more auditory (Fig. 4). It is possible that this arrangement reduces the number of crossing fibers between parietal and prefrontal brain regions dedicated to the same modality. Like pages in a book, which are stacked then folded around a central crease, the furthest posterior regions can project to the furthest anterior regions without crossing frontoparietal projections in between. “Vertical” (ventral–dorsal) gradients were observed in similar directions in both PL and PFC.

It is important to note that regions exhibiting a strong preference for one modality may still be involved in processing information in the other. This appears to be the case in the FEF, which have strong preference for visual cortices, but also show frequency‐locked activity to attended sounds [Bharadwaj et al., 2014]. The present results imply that these regions may be predominantly concerned with a given modality, even if they display multimodal properties. Although a graded dorsal–ventral pattern of visual–auditory preference was observed, it is possible that there are distinct auditory and visual loci in frontoparietal cortex. For example, in the PL visual structural connections seemed to be clustered around a dorsal center (Fig. 4). As the reported analyses were conducted at the group‐level, one possibility is that the gradients were a consequence of averaging across subjects. However, when individual subjects were tested in the present analysis, qualitatively similar results were observed. Furthermore, although across‐subject averaging might explain the gradual change from visual to auditory connectivity, such a scenario would still involve a strong visual bias in dorsal, and auditory bias in ventral frontoparietal cortex. Previous studies compliment the finding of a graded transition, with a multimodal intraparietal sulcus localized between dorsal–visual and ventral–auditory parietal regions [Bremmer et al., 2001; Langner et al., 2011]. Similar gradient transitions have also been reported between different functional networks [Anderson et al., 2011].

In the PL, the vertical and horizontal functional gradients were largely explained by the relative distance to the targets (Fig. 5). Functional gradients in the PFC were largely unaffected by this covariate. Conversely in the PFC, the structural gradients were almost entirely explained by the relative distance to the targets (apart from the horizontal gradient in the left PFC), whereas the PL gradients were largely unaffected. This raises the possibility, particularly in the tractography results, that the gradients detected were due to artifactual causes. However, it is important to note that an overlap between relative distance and relative connectivity does not mean that the connectivity differences are nonexistent. It is possible that the connectivity patterns in frontoparietal cortices evolved precisely as a consequence of the relative position of the region in relation to the sensory cortices.

Another possibility is that the observed results were a consequence of the choice of using ROIs constrained (approximately) to early sensory regions. The parietal and frontal cortices are typically considered to be higher‐order regions, and their function may be more defined by areas higher up the sensory hierarchy (e.g., V3 or planum temporale) than areas within Heschl's gyrus and the intracalcarine sulcus. In resting state FC, visual regions V1, V2, and V3 typically show correlated activity [Raemaekers et al., 2014; Smith et al., 2004; Yeo et al., 2011]. Thus, the functional results observed are unlikely to be a consequence of the choice of ROIs. When we used extended resting‐state‐derived auditory and visual networks as the target ROIs, gradients were still observed in both frontal and parietal regions. These larger ROIs extended into the parietal lobe, and so were not included in the present study to avoid biasing the connectivity results. However, the similar findings with these networks‐defined ROIs indicate that the graded functional pattern was not solely due to the choice of ROI. One possibility, that was not tested in the present analysis, is that the structural connections from the parietal and frontal lobe are indeed more weighted toward intermediate sensory structures. If that were the case, it is possible that the auditory gradient observed would be dominated by strong visual connections in ventral frontoparietal regions. At least in the functional case, the present findings suggest this is not the case. Finally, it is possible that there are inter‐hemispheric differences in the connectivity between sensory regions and frontoparietal cortices (e.g., due to the lateralization of language function) that were not tested here given the use of bilateral ROIs in the functional analysis.

In conclusion, in this study we report the existence of striking asymmetries in the relative connectivity profile of frontoparietal cortex with visual and auditory cortices. We propose that these asymmetries may represent the different roles that activity in frontoparietal control networks can take during different cognitive tasks. Specifically, we propose that dorsal frontoparietal activity is more likely to represent visual processes, while ventral frontoparietal activity is more likely to represent auditory processing. This work adds weight to recent theories that auditory and visual attention are subserved by separable dorsal and ventral networks [Braga et al., 2013b; Braga et al., 2016; Degerman et al., 2006; Kong et al., 2014; Salmi et al., 2007; Seydell‐Greenwald et al., 2014] and suggests that further work is necessary to disentangle the precise role of frontoparietal activity in multimodal processing.

## Supporting information

Supporting Information
