# Supplementary material for: Auditory and visual connectivity gradients in frontoparietal cortex
Source: Hum Brain Mapp. 2016 Aug 29;38(1):255–70. doi: 10.1002/hbm.23358 (PMC5215394; doi:10.1002/hbm.23358)
Supplement: Supplementary file 1 — Supporting Information [file HBM-38-255-s001.docx]

**Human Brain Mapping – Supplemental Materials**

**Title:**

Auditory and visual connectivity gradients in frontoparietal cortex

**Short title:**

Frontoparietal audiovisual gradients

**Authors:**

Rodrigo M. Braga^1,2,3^, Peter J. Hellyer^3,4^, Richard J. S. Wise^3^ & Robert Leech^3^

**Author Affiliation:**

^1^Center for Brain Science, Harvard University, Cambridge, Massachusetts, USA.

^2^Athinoula A. Martinos Center for Biomedical Imaging, Department of Radiology, Massachusetts General Hospital & Harvard Medical School, Charlestown, Massachusetts, USA.

^3^The Computational, Cognitive and Clinical Neuroimaging Laboratory, Division of Brain Sciences, Hammersmith Hospital Campus, Imperial College London, London, UK.

^4^Centre for Neuroimaging Sciences, Institute of Psychiatry, Psychology & Neuroscience, King’s College London.

**Corresponding Authors:**

Rodrigo M. Braga

A: Center for Brain Science, Harvard University, Cambridge, Massachusetts, USA

E: rbraga@fas.harvard.edu

**Supplemental Figure 1: Group-averaged anatomical region of interest(ROI) for auditory cortex back-projected onto each individual’s anatomical image.**

**
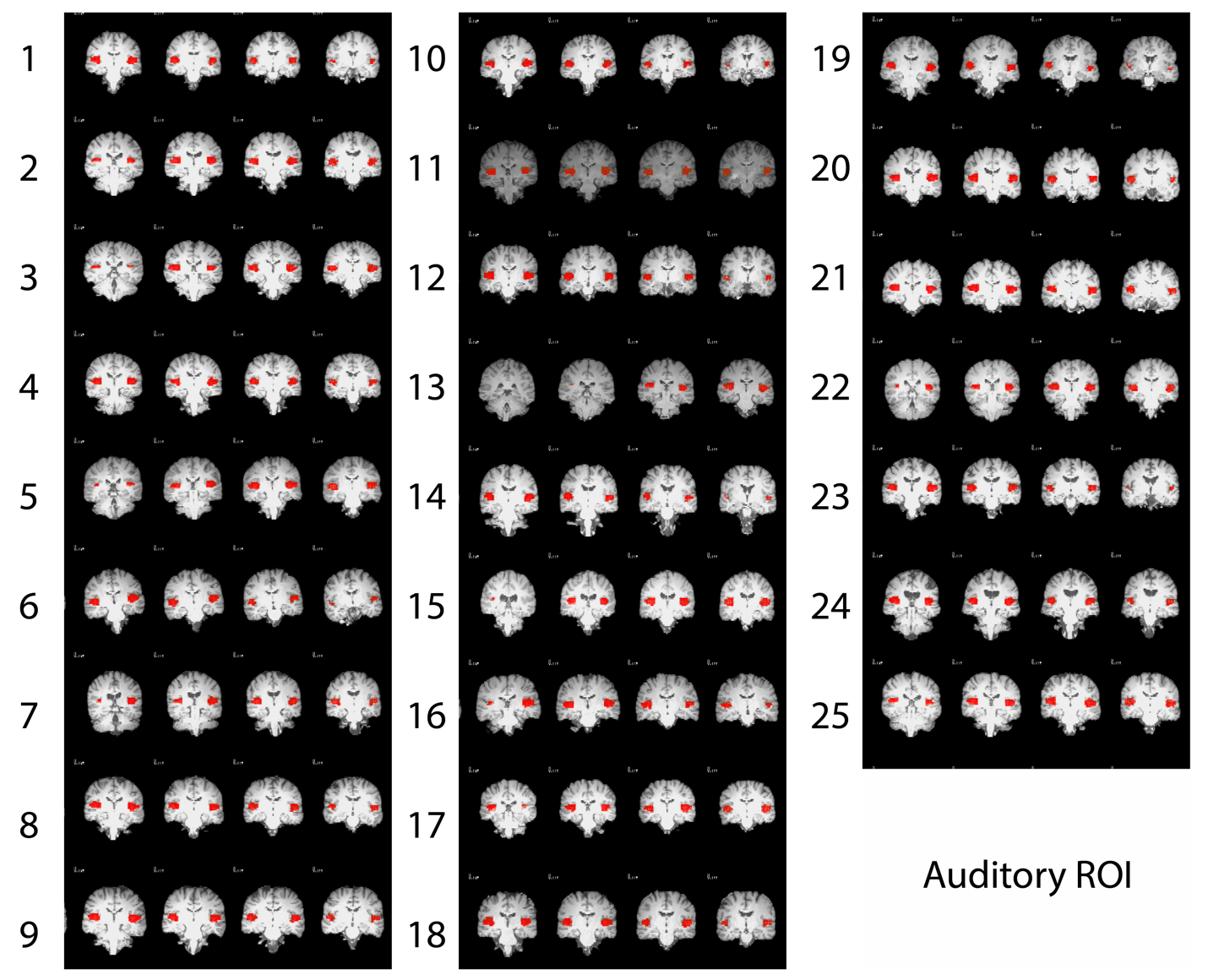
**

**Supplemental Figure 2: Group-averaged anatomical region of interest (ROI) for visual cortex back-projected onto each individual’s anatomical image.**

**
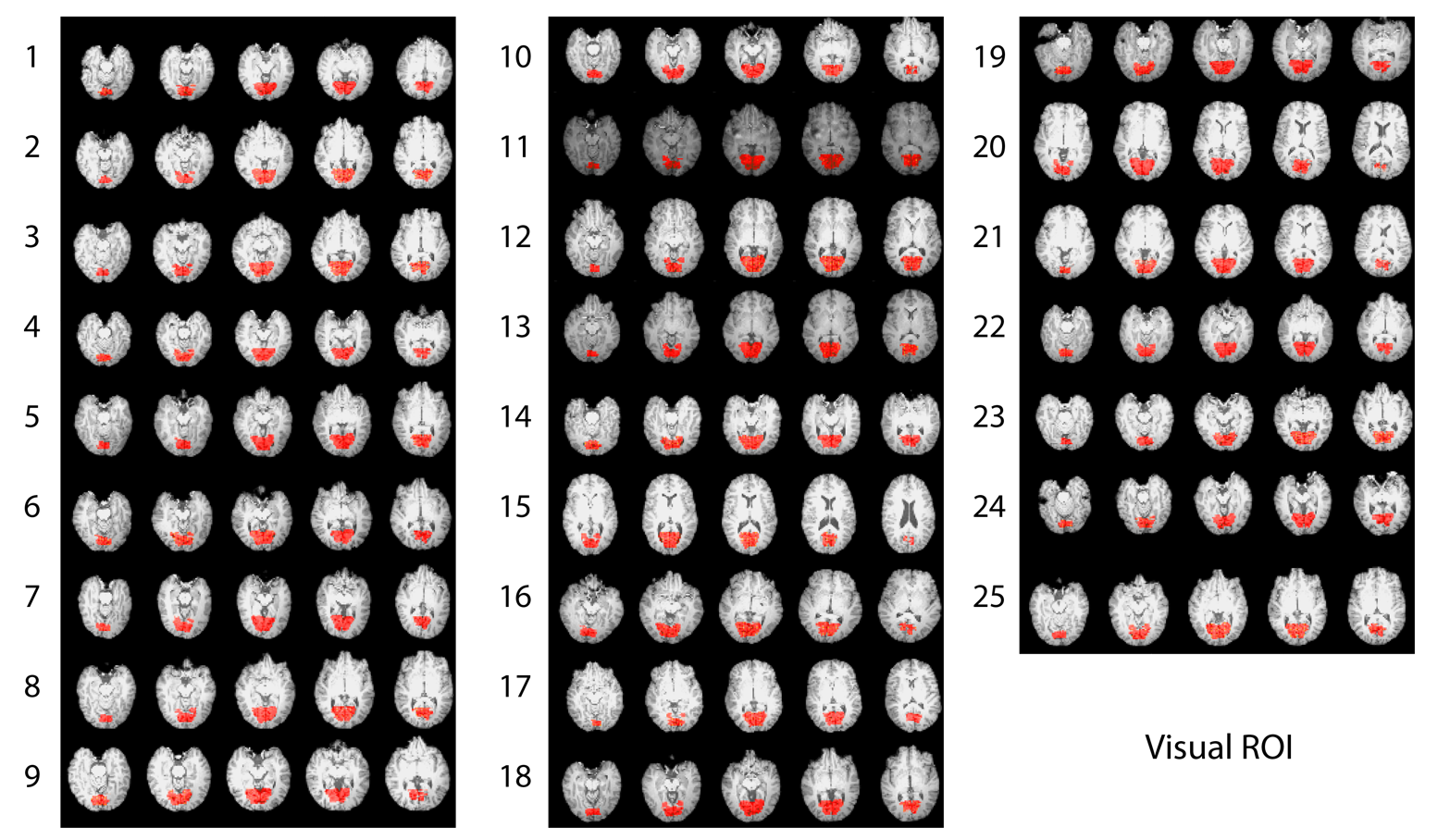
**
